# Extracorporeal Life Support for Cardiogenic Shock in Octogenarians: Single Center Experience

**DOI:** 10.3390/jcm12020585

**Published:** 2023-01-11

**Authors:** Nadezda Scupakova, Karolis Urbonas, Agne Jankuviene, Lina Puodziukaite, Povilas Andrijauskas, Vilius Janusauskas, Aleksejus Zorinas, Kestutis Laurusonis, Pranas Serpytis, Robertas Samalavicius

**Affiliations:** 1 II Department of Anaesthesiology and Intensive Care, Vilnius University Hospital Santaros Klinikos, LT-08661 Vilnius, Lithuania; 2 Clinic of Emergency Medicine, Institute of Clinical Medicine, Faculty of Medicine, Vilnius University, LT-08661 Vilnius, Lithuania; 3 Clinic of Cardiovascular Diseases, Institute of Clinical Medicine, Faculty of Medicine, Vilnius University, LT-08661 Vilnius, Lithuania; 4 Kardiolitos Klinikos, LT-08661 Vilnius, Lithuania

**Keywords:** extracorporeal membrane oxygenation, ECMO, post-cardiotomy, cardiac surgery, cardiogenic shock, elderly, octogenarians, septuagenarians

## Abstract

Background: The age limit for the use of extracorporeal membrane oxygenation (ECMO) support for post-cardiotomy cardiac failure is not defined. The aim of the study was to evaluate the outcomes of octogenarians supported with ECMO due to cardiogenic shock. Methods: A retrospective review of consecutive elderly patients supported with ECMO during a 13-year period in a tertiary care center. Patient’s demographic variables, comorbidities, perioperative data and outcomes were collected from patient medical records. Data of octogenarian patients were compared with the septuagenarian group. The main outcomes of the study was in hospital mortality, 6-month survival and 1-year survival after hospital discharge and discharge options. Multivariate logistic regression analysis was performed to identify the factors associated with hospital survival. Results: Eleven patients (18.3%) in the elderly group were octogenarians (aged 80 years or above), and forty-nine (81.7%) were septuagenarians (aged 70–79 years). There were no differences except age in demographic and preoperative variables between groups. Pre ECMO SAVE, SOFA, SAPS—II and inotropic scores were significantly higher in septuagenarians than octogenarians. There was no statistically significant difference in hospital mortality, 6-month survival, 1 year survival or discharge options between groups. Conclusions: ECMO could be successfully used in selected octogenarian patients undergoing cardiac surgery to support a failing heart. An early decision to initiate ECMO therapy in elderly post-cardiotomy shock patients is associated with favorable outcomes.

## 1. Introduction

Post-cardiotomy cardiogenic shock is a life-threatening complication that occurs in 0.2–6% of patients undergoing cardiac surgery [1]. Despite advances in patient management, the mortality rate of cardiogenic shock remains high in this patient population and may reach up to 70% [2]. Intra-aortic balloon pump (IABP), once used as the first-line mechanical assist device to support failing hearts in the case of severe cardiogenic shock has been proven to be ineffective [3]. Nowadays, the use of extracorporeal membrane oxygenation (ECMO) has become one of the most utilized mechanical circulatory support (MCS) devices to maintain adequate cardiac output. During the last two decades, the use of ECMO in the treatment of post-cardiotomy shock has progressed considerably, the technology has improved and the experience of institutions has increased [2]. Irrespective of the age of the patients, encouraging data are emerging on improved early and late survival with the use of extracorporeal life support (ECLS) [4].

The world’s population is ageing, and half of the patients referred for cardiac surgery nowadays are elderly [5]. By 2050, one in four persons living in Europe and North America could be aged 65 or over, and the number of persons aged 80 years or over is projected to triple [6]. The percentage of elderly patients in need of cardiac surgery will only increase, as well as the expectations of patients and their families for a good outcome. Currently, there is no clear consensus on the age limit for the use of ECMO support for refractory post-cardiotomy cardiogenic shock.

The aim of the study was to evaluate the outcomes of octogenarians supported with ECMO due to cardiogenic shock following cardiac surgery and compare them with the younger patient group.

## 2. Materials and Methods

In this study, we reviewed our 13 years of experience with ECMO use to support the failing heart after cardiac surgery in elderly patients in a single tertiary hospital. Our study was approved by the Vilnius Regional Bioethics Committee (Lithuania), reference number 158200-16-850-259. Sixty patients older than 70 years supported with ECMO following cardiac surgery were included in the analysis. The indication for support was the inability to wean the patient from cardiopulmonary bypass or post-bypass cardiac failure, unresponsive to medical treatment and the use of IABP. If ECMO support was initiated during surgery, intra-thoracic cannulation was used. In the case of post-operative cardiac failure, the decision for intra-thoracic or peripheral cannulation was made by the surgeon and anesthesiologist in charge. If femoral arterial cannulation was performed, a distal perfusion catheter (8 Fr) was placed in the femoral artery or a 4 Fr catheter in the posterior tibial artery for retrograde perfusion. In all cases, a near-infrared spectroscopy (Invos 5100, Medtronic Inc, Minneapolis, MN, USA) monitor was used to evaluate the adequacy of distal extremity perfusion. ECMO flows were maintained to keep mixed oxygen saturation greater than 60%. Heparin infusion was initiated when drainage from the chest tubes was minimal and there were no signs of bleeding. The targeted activated clotting time (ACT) level was 180–220 s. The target for hemoglobin level was above 80 g/L. In patients with no signs of bleeding, platelet count was maintained greater than 40,000 cells/mm^3^, while in the patients with a clear blood loss, platelet count was aimed to be maintained above 100,000 cells/mm^3^. Fresh frozen plasma, clotting factor concentrates or cryoprecipitate transfusions were performed if needed. Weaning from ECMO support was considered at least following 48 h of support with the confirmed recovery of cardiac function based on cardiac ultrasound and hemodynamic evaluation. Aortic velocity time integral (VTI) ≥ 12 cm and left ventricle ejection fraction (LVEF) of more than 20–25% at an ECMO flow of 2 L/min were considered as signs of myocardial recovery. 

Patients were assigned into two groups according to their age: patients from 70 to 79 years (septuagenarians) and from 80 to 89 years (octogenarians). Patient’s demographic variables, comorbidities, pre-existing medical conditions, risk score evaluations (SAVE, SOFA, SAPS-II, VIS, Frailty, Euroscore II), perioperative data and outcomes were collected from medical records. The outcomes were in-hospital mortality, 6-month survival after hospital discharge, 1-year survival after hospital discharge and discharge options. These included discharge home (directly or after rehabilitation in the same hospital) or “non–home discharge” (NHD). NHD is described as discharge to another hospital, nursing home, palliative care, chronic care facility or other locations patients are transferred due to their impaired functions and inability to be discharged home.

### Statistical Analysis

Statistical analysis was performed using the software package SPSS 23.0 (IBM Corp., Armonk, NY, USA). The quantitative normality of continuous data was evaluated using the criteria of histograms, rectangular diagrams and the Shapiro–Wilk test (*p* > 0.05). The quantitative continuous data, distributed as normal, presented as the mean ± standard deviation. The quantitative continuous data distributed outside the normal distribution are presented as the median and quartile intervals. The categorical data were expressed as percentages. Categorical variables (between patients who survived to hospital discharge and those who did not) were compared using the χ^2^ or Fisher criterion. The Mann–Whitney–Wilcoxon test was used to compare quantitative continuous data. A *p*-value < 0.05 was considered statistically significant. Univariate logistic regression analysis of risk factors was performed and variables with *p*-value of less than 0.1 were included in multivariate regression analysis. A *p*-value < 0.05 was considered statistically significant.

## 3. Results

Between 1 November 2008 and 1 July 2021, 249 veno-arterial (VA) ECMO procedures were performed at our center. Sixty patients (24%) were 70 years old or older and were included in the final analysis as the “elderly group”. Eleven patients (18.3%) in the elderly group were octogenarians (aged 80 years or above), and forty-nine (81.7%) were septuagenarians (aged 70–79 years). Groups were compared with each other. There was no significant difference except age between groups. The baseline patient’s characteristics and preoperative data are shown in Table 1.

There was no difference in perioperative data between groups. The types of surgical procedures data are summarized in Table 2.

In the majority of cases VA ECMO was initiated in the intensive care unit (N = 37, 61.7%), and 23 cases (38.3%) were started in the operating room. There were 47 cases of central (78.3%) and 13 cases of peripheral VA ECMO (21.7%). Central cannulation was more common in the septuagenarian group than the octogenarian group (83.7% vs. 54.5%, respectively, *p* = 0.034). The median duration of the ECMO support was 148 (71–288) h and was longer in the septuagenarian group than the octogenarian group at 178 (92–300) h vs. 70 (46–93) h, respectively, (*p* = 0.007).

Pre-ECMO SAVE, SOFA, SAPS–II and VIS scores were significantly higher in septuagenarians than octogenarians. Pre-ECMO data are shown in Table 3.

Thirty-one patients (52%) were successfully weaned from ECMO, and 13 (22%) patients survived hospital discharge. There was no statistically significant difference in hospital mortality between the groups (7 (64%) vs. 40 (82%), (*p* = 0.19)). Out of 13 discharged patients, 11 were discharged home and 2 were transferred to another hospital for extended care. All of the octogenarian group patients and 22% of the septuagenarians experienced NHD. Discharge options did not statistically differ between octogenarian and septuagenarian patients. All patients except one (92.3%) were still alive at 6 months after discharge from the hospital and overall 1-year survival reached 84.6%. There also was no statistically significant difference between the groups in survival after hospital discharge. Bleeding events on ECMO were more common in septuagenarians than octogenarians (43 (88%) vs. 4 (36%), respectively, (*p* = 0.001) and were associated with higher resternotomy and blood product transfusion rates in the septuagenarian group. Patient outcomes and complications are summarized in Table 4.

Multivariate logistic regression model was performed to assess the independent association between age and hospital discharge, including co-variables, which had a p-value of less than 0.1 in the univariate analysis (vasoactive–inotropic score, acute renal failure pre-ECMO, mean arterial pressure pre-ECMO and cardiac arrest), or were considered to be important factors by investigators (intubation duration pre-ECMO). Shorter intubation time pre-ECMO was the only significant predictor of the favorable outcome (*p* = 0.023, 95% CI = 1.025–1.384). Variables included in multivariate analysis are presented in Table 5.

## 4. Discussion

Globally, during the last decade, ECLS has been increasingly utilized to support patients with cardiogenic shock. The in-hospital mortality tended to decrease over time; however, the older patients were more likely not to survive to hospital discharge [7]. Veno-arterial ECMO might support patients for days or weeks until successful weaning after recovery of cardiac function as a bridge to heart transplantation or the implantation of a long-term mechanical assist device. The use of ECMO until successful weaning might be the only option in octogenarians or other elderly patients. The majority of centers have age limits for orthotopic heart transplantation or implantation of a durable left ventricle assist device (LVAD). Patients older than 65 years, undergo orthotopic heart transplantation or receive LVADs significantly less frequently compared to the younger population [8]. Due to those reasons, the recovery of cardiac function with the following weaning from support seems to be the only option for the elderly patients.

Results of our study show that there were no significant differences in preoperative characteristics between the septuagenarians and octogenarians supported with ECMO for cardiac failure in our institution. However, some preoperative characteristics, such as decreased mobility, rate of preoperative renal impairment, diabetes or recent MI, were more frequently seen in the younger patient group, and significance might not be reached due to the small sample size. Before the initiation of ECMO, SAVE score, SOFA score, SAPS II score and vasoactive inotropic score were statistically significantly higher in the septuagenarian group, clearly showing that patients in this group were in the worst condition compared to the octogenarian group before ECMO initiation. This might be biased by decision making, when fragile and morbid octogenarians might be refused support with ECMO. The decision to initiate ECMO or to proceed with more conservative treatment in older septuagenarians and octogenarians was made after careful evaluation of the physical status rather than based on the age itself. Recently published studies have shown that advanced age should not disqualify patients from cannulating and supporting them with VA ECMO, and a survival rate of almost 43% can be achieved in this group of patients [9]. The delay in the initiation of ECMO in patients with cardiogenic shock leads to poor survival, and it is especially important in elderly patients. The duration of ECMO support is also important, especially in patients who cannot be candidates for the transplantation or implantation of durable LVAD. Three to four days should be sufficient for myocardium to recover from myocardium stunning according to previous studies [10,11]. In our study, the duration of ECMO support for octogenarians who survived was 47 ± 27 h and 133 ± 167 h for non-survivors. There was a statistically significant difference in the duration of ECMO support between octogenarians and the younger patient group in our study. This might be determined by decision making, for example, the withdrawal of support in older patients if there were no signs of myocardial recovery. Our data confirm the data of previous investigators that show 3–4 days of support would be optimal in the elderly who are not eligible for the durable assist devices implantation.

Bleeding events on ECMO occurred more frequently in septuagenarians than octogenarians and were associated with higher resternotomy and blood product transfusion rates in the septuagenarian group. This might be due to more severe cardiogenic shock or a more severe condition before ECMO initiation of the younger patient group. Higher blood loss and extreme transfusion requirement were associated with increased mortality in VA-ECMO-supported patients [12].

Life expectancy is increasing in developed countries, and inequality in life expectancy in different countries is rising accordingly. Life expectancy has considerably increased in most developed countries during the recent years [13]. It is believed that life expectancy is influenced by social, economic and health indicators [14]. These extreme health inequities partly reflect wealth inequalities between countries as wealthier countries have a higher average life expectancy than poorer countries. It is worth mentioning that even across Europe, a wide variation in life expectancy is present, and less favorable mortality trends in Eastern Europe show health care problems and a failure to implement effective health care policies [15]. As there are differences in healthy life expectancy between countries, the comparison of a critically ill patient population remains controversial. For patients for whom post-cardiotomy cardiac failure is felt to be reversible, advanced age is a relative contraindication and the goals of treatment should be considered [16]. Statistically significant higher lactate level before ECMO initiation was found in the septuagenarian patient group. High lactate level before the initiation of ECMO support was shown to be an important prognostic factor of unfavorable outcomes in the elderly population [17,18]. Multivariate analysis revealed that intubation time before ECMO initiation was a significant predictor of hospital survival. This proves that the decision to initiate ECMO support should be considered at the early onset of severe post-cardiotomy shock in the elderly patients. Despite lower hospital survival rates among the elderly, patients who survive to discharge have an acceptable long-term survival [18,19], and despite the complex clinical course, they do have satisfactory mental and physical recovery [20]. Eleven of our patients (85%) were discharged home and two (15%) were transferred to another hospital for extended medical care. NHD is a composite measure with an individual patient-centered outcome, and it is associated with patient disability or impaired organ function, which means they are not allowed to discharge the patients to their homes. In a recent study, NHD after cardiac surgery was found to be 7% [21] and was associated with decreased overall survival.

The crucial limitation of our study is not analyzing the quality of life in elderly patients who survived to hospital discharge. The retrospective design of the study and small sample size at single institution are also the limitations of the study. Large multicenter prospective studies and the evaluation of long-term survival and quality of life might provide a definitive answer on the decision making of elderly patients by selecting them as candidates for ECMO support.

## 5. Conclusions

In conclusion, the age alone should not be considered as a contraindication for MCS. In selected patients, ECMO support can be successfully used for developing cardiogenic shock. Further prospective multicenter studies are needed to identify patients who would benefit the most.

## Figures and Tables

**Table 1:** Baseline patient’s characteristics and preoperative data.

Clinical Variables	Overall, N = 60	Octogenarians, N = 11	Septuagenarians, N = 49	*p* Value
Age, years	75 (72–78)	82 (81–83)	74 (72–77)	0.00001
Gender, male	40 (67)	6 (55)	34 (69)	0.345
BSA, m^2^	1.95 (1.75–2.04)	1.78 (1.7–1.9)	1.96 (1.8–2.1)	0.413
Diabetes on insulin	7 (12)	0 (0)	7 (14)	0.117
Diabetes mellitus	17 (28)	1 (9)	16 (33)	0.288
CCS class 4 angina	12 (20)	4 (36)	8 (16)	0.133
Critical preoperative state	17 (28)	5 (45)	12 (24)	0.163
Preoperative LVEF, %	45 ± 13	40 (35–47)	50 (40–55)	0.168
COPD	6 (10)	0 (0)	6 (12)	0.221
NYHA III	41 (68)	6 (55)	35 (71)	0.277
NYHA IV	19 (32)	5 (45)	14 (29)	0.277
Previous cardiac surgery	12 (20)	1 (9)	11 (22)	0.317
Poor mobility	4 (7)	0 (0)	4 (8)	0.327
Creatinine clearance, mL/min	60 ± 22	56 (45–62)	59 (45–78)	0.408
Chronic renal failure	14 (23)	1 (9)	13 (27)	0.217
Congenital heart disease	2 (3)	0 (0)	2 (4)	0.496
Recent MI	17 (28)	4 (36)	13 (27)	0.513
PAP > 55 mmHg	14 (23)	0 (0)	14 (29)	0.693
Extracardiac arteriopathy	15 (25)	3 (27)	12 (24)	0.847
Frailty score	5 ± 1	4 (4–5)	5 (4–5)	0.293
EuroSCORE II	13 ± 16	8.5 (4.9–17.3)	6.6 (3.3–14.3)	0.585

Continuous variables are presented as means ± standard deviation or median (Q1–Q3). Categorical variables are presented as counts and percentages. BSA—body surface area, LVEF—left ventricular ejection fraction, COPD—chronic obstructive pulmonary disease, NYHA—New York Heart Association heart failure classification system, MI—myocardial infarction, PAP—pulmonary artery pressure, EuroSCORE—European System for Cardiac Operative Risk Evaluation.

**Table 2:** Type of surgical procedures and perioperative data.

Clinical Variables	Overall, N = 60	Octogenarians, N = 11	Septuagenarians, N = 49	*p* Value
Duration of surgery, min	320 (240–468)	308 (225–363)	320 (265–490)	0.332
CPB time, min	189 (137–254)	215 (164–282)	186 (134–247)	0.356
Aortic cross clamp time, min	109 ± 44	106 (85–134)	105 (75–135)	0.764
Urgent surgery	17 (28)	3 (27)	14 (29)	0.931
CABG	15 (25)	2 (18)	13 (27)	0.563
AVR	12 (20)	2 (18)	10 (20)	0.868
CABG and valve	7 (12)	2 (18)	5 (10)	0.456
Double valve	12 (20)	2 (18)	10 (20)	0.462
Other procedures	14 (23)	3 (27)	11 (22)	0.732

Continuous variables are presented as means ± standard deviation or median (Q1–Q3). Categorical variables are presented as counts and percentages. CPB—cardiopulmonary bypass, CABG—coronary artery bypass graft, AVR—aortic valve replacement.

**Table 3:** Pre-ECMO data.

Clinical Variables	Overall, N = 60	Octogenarians, N = 11	Septuagenarians, N = 49	*p* Value
Lactate pre-ECMO, mmol/L	11 ± 7	5.27 (4.1–8.5)	10.02 (5.9–15.2)	0.034
ARF pre-ECMO	33 (55)	3 (27)	30 (61)	0.041
MAP pre-ECMO, mmHg	45 ± 14	46 (43–65)	43 (37–50)	0.047
MV pre-ECMO, h	13 (8–32)	8 (4-31)	17 (9–32)	0.172
IABP prie-ECMO	43 (72)	7 (64)	36 (73)	0.513
SAVE score, points	−10 ± 6	−5 (−7.5–−2.5)	−11 (−15–−6)	0.002
SOFA score, points	9 ± 3	7 (5–7.5)	8 (7–11)	0.009
SAPS-II score, points	46 ± 11	40 (35–42.5)	47 (38–54)	0.012
VIS score, points	60 ± 31	42 (27.5–57.3)	60 (47.8–72.8)	0.045

Continuous variables are presented as means ± standard deviation or median (Q1–Q3). Categorical variables are presented as counts and percentages. ARF—acute renal failure, ECMO—extracorporeal membrane oxygenation, MAP—mean arterial pressure, MV—mechanical ventilation, IABP—intra-aortic balloon pump, SAVE—survival after veno-arterial ECMO, SOFA—sequential organ failure assessment, SAPS—simplified acute physiology score, VIS—vasoactive–inotropic score.

**Table 4:** Patient outcomes and complications.

Clinical Variables	Overall, N = 60	Octogenarians, N = 11	Septuagenarians, N = 49	*p* Value
In-hospital mortality	47 (78)	7 (64)	40 (82)	0.19
Weaned from ECMO	31 (52)	5 (45)	26 (53)	0.648
Death after weaning	18 (30)	1 (9)	17 (35)	0.094
Hospital discharge	13 (22)	4 (36)	9 (18)	0.133
Home discharge	11 (18)	4 (36)	7 (14)	0.087
NHD	2 (3)	0 (0)	2 (4)	0.496
6-month survival AHD	12 (20)	4 (36)	8 (16)	0.133
1-year survival AHD	11 (18)	3 (27)	8 (16)	0.396
ICU stay, days	14 (9–21)	13 (5–15)	15 (9–21)	0.151
Hospital stay, days	24 (15–37)	15 (13–37)	24 (17–37)	0.395
Bleeding on ECMO	47 (78)	4 (36)	43 (88)	0.001
Surgical site bleeding	31 (52)	1 (9)	30 (61)	0.002
Cannulation site bleeding	26 (43)	3 (27)	23 (47)	0.234
Gastrointestinal bleeding	9 (15)	2 (18)	7 (14)	0.744
Other bleeding	22 (37)	1 (9)	21(43)	0.036
Resternotomy	38 (63)	4 (36)	34 (69)	0.037
RBC, units	28 ± 14	8 (4–10)	21 (14–31)	0.028
FFP, units	18 ± 14	8 (5–24)	16 (9–25)	0.608
Platelets, units	11 ± 8	9 (6–13)	10 (4–15)	0.974
RRT	42 (70)	5 (45)	37 (76)	0.049
Infection	37 (62)	5 (45)	32 (65)	0.221
Neurological complications	13 (22)	2 (18)	11 (22)	0.756

Continuous variables are presented as means ± standard deviation or median (Q1–Q3). Categorical variables are presented as counts and percentages. ECMO—extracorporeal membrane oxygenation, ICU—intensive care unit, RBC—red blood cells, FFP—fresh frozen plasma, RRT—renal replacement therapy, AHD—after hospital discharge, NHD—non–home discharge.

**Table 5:** Multivariate analysis of factors for hospital discharge.

Factor	Odds Ratio		*p* Value
	Estimate	95% CI	
	Multivariate		
Age	1.141	0.909–1.432	0.255
VIS score	1.014	0.989–1.042	0.332
ARF pre-ECMO	0.710	0.141–3.585	0.678
MAP pre-ECMO	0.971	0.916–1.028	0.309
Cardiac arrest pre-ECMO	0.492	0.064–3.780	0.495
MV duration pre-ECMO	1.191	1.025–1.384	0.023

VIS—vasoactive–inotropic score, ARF—acute renal failure, MAP—mean arterial pressure, MV—mechanical ventilation, ECMO—extracorporeal membrane oxygenation.

## References

[1] Sylvin E.A., Stern D.R., Goldstein D.R. (2010). Mechanical Support for Postcardiotomy Cardiogenic Shock: Has Progress Been Made?. J. Card. Surg..

[2] Biancari F., Perroti A., Dalen M., Guerrieri M., Fiore A., Reichart D., Dell’Aquila A.M., Gatti G., Ala-Kokko T., Kinnunen E.M. (2018). Meta-analysis of the outcome after postcardiotomy venoarterial extracorporeal membrane oxygenation in adult patient. J. Cardiothorac. Vasc. Anesth.

[3] Thiele H., Zeymer U., Neumann F.J., Ferenc M., Olbrich H.G., Hausleiter J., Richardt G., Hennersdorf M., Empen K., Fuernau S. (2012). Intraaortic balloon support for myocardial infarction with cardiogenic shock. N. Engl. J. Med..

[4] Schaefer A.K., Riebandt J., Bernardi M.H., Distelmaier K., Goliasch G., Zimpfer D., Laufer G., Wiedemann D. (2022). Fate of patients weaned from postcardiotomy extracorporeal life support. Eur. J. Cardiothorac. Surg..

[5] Afilalo L., Eisenberg M.J., Morin J.F., Bergman H., Monette J., Noiseux N., Perrault L.P., Alexander K.P., Langlois Y., Dendukuri N. (2010). Gait speed as an incremental predictor of mortality and major morbidity in elderly patients undergoing cardiac surgery. J. Am. Coll. Cardiol..

[6] United Nations World Population Prospects: The 2019 Revision. https://population.un.org/wpp/.

[7] Karagiannidis C., Brodie D., Strassmann S., Stoelben E., Phillip A., Bein T., Muller T., Windisch W. (2016). Extracorporeal membrane oxygenation: Evolving epidemiology and mortality. Intensive Care Med..

[8] Chouairi F., Vallabhajosyula S., Mullan C., Mori M., Geirsonn A., Desai N.R., Ahmad T., Miller P.E. (2020). Transition to advanced therapies in elderly patients supported by extracorporeal membrane oxygenation therapy. J. Card. Fail..

[9] Armas I.A.S., Holifield L., Janowiak L.M., Akay M.H., Patarroyo M., Nescimbene A., Akkanti B.H., Patel M., Patel J., Marcano J. (2022). The use of veno-arterial extracorporeal membrane oxygenation in the octogenarian population: A single-center experience. Perfusion.

[10] Saxena P., Neal J., Joyce L.D., Greason K.L., Schaff H.V., Guru P., Shi W.Y., Burkhart H., Li Z., Oliver W.C. (2015). Extracorporeal membrane oxygenation support in postcardiotomy elderly patients: The Mayo Clinic experience. Ann. Thorac. Surg..

[11] Rastan A.J., Lachmann N., Walther T., Doll N., Gradistanac T., Gommert J.F., Lehmann S., Wittekind C., Mohr F.W. (2006). Autopsy findings in patients on postcardiotomy extracorporeal membrane oxygenation (ECMO). Int. J. Artif. Organs..

[12] Guimbretiere G., Anselmi A., Roisne A., Lelong B., Corbineau H., Langanay T., Flecher E., Verhoye J.P. (2019). Prognostic impact of blood product transfusion in VA and VV ECMO. Perfusion.

[13] Liou L., Joe W., Kumar A., Subramanian S.V. (2020). Inequalities in life expectancy: An analysis of 201 countries, 1950–2015. Soc. Sci. Med..

[14] Freeman T., Gesesew H.A., Bambra C., Giugliani E.R.J., Popay J., Sanders D., Macinko J., Musolino C., Baum F. (2020). Why do some countries do better or worse in life expectancy relative to income? An analysis of Brazil, Ethiopia, and the United States of America. J. Equity Health.

[15] Mackenbach J.P., Karanikolos M., McKee M. (2013). The unequal health of Europeans: Successes and failures of policies. Lancet.

[16] Lorusso R., Whitman G., Milojevic M., Raffa G., McMullan D.M., Boeken U., Haft J., Bermudez C.A., Shah A.S., D’Alessandro D.A. (2021). 2020 EACTS/ELSO/STS/AATS expert consensus on post-cardiotomy extracorporeal life support in adult patient. Ann. Thorac. Surg..

[17] Samalavicius R., Norkiene I., Scupakova N., Sabliauskas J., Urbonas K., Andrijauskas P., Jankuviene A., Puodziukaite L., Zorinas A., Janusauskas V. (2020). Evaluation of risk factors for adverse outcome in extracorporeal membrane oxygenation-supported elderly postcardiotomy patients. Perfusion.

[18] Biancari F., Saeed D., Fiore A., Dalen M., Ruggieri V.G., Jonsson K., Gatti G., Zipfel S., Dell’Aquila A.M., Chocron S. (2019). Postcardiotomy venoarterial extracorporeal membrane oxygenation in patients aged 70 years or older. Ann. Thorac. Surg..

[19] Narotsky D.L., Mosca M.S., Mochari-Greenberger H., Beck J., Liao M., Mongero L., Bacchetta M. (2016). Short-term and long-term survival after veno-arterial extracorporeal membrane oxygenation in adult patient population: Does the olde age matter. Perfusion.

[20] Norkiene I., Jovaisa T., Scupakova N., Janusauskas V., Rucinskas K., Serpytis P., Laurusonis k., Samalavicius R. (2019). Long-term quality of life in patients treated with extracorporeal membrane oxygenation for postcardiotomy cardiogenic shock. Perfusion.

[21] Ramanan M., Kumar A., Anstey C., Shekar K. (2021). Non-home discharge after cardiac surgery in Australia and New Zealand: A cross-sectional study. BMJ Open.

